# The association between antenatal coffee consumption and preeclampsia: a systematic review and meta-analysis

**DOI:** 10.1265/ehpm.24-00149

**Published:** 2024-09-21

**Authors:** Ahmed Arafa, Masayuki Teramoto, Haruna Kawachi, Chisa Matsumoto, Saya Nosaka, Miki Matsuo, Yuka Yasui, Yuka Kato, Yoshihiro Kokubo

**Affiliations:** 1 Department of Preventive Cardiology, National Cerebral and Cardiovascular Center, Suita, Japan; 2 Department of Public Health, Faculty of Medicine, Beni-Suef University, Beni-Suef, Egypt; 3 Department of Environmental Medicine and Population Sciences, Graduate School of Medicine, Osaka University, Suita, Japan; 4 Department of Cardiology, Center for Health Surveillance and Preventive Medicine, Tokyo Medical University Hospital, Shinjuku, Japan; 5 Department of Hypertension and Nephrology, National Cerebral and Cardiovascular Center, Suita, Japan; 6 Graduate School of Human Life and Science, Doshisha Women’s College of Liberal Arts, Kyoto, Japan; 7 Division of Health Sciences, Osaka University Graduate School of Medicine, Suita, Japan; 8 School of Cardiovascular & Metabolic Health, University of Glasgow, Scotland, Glasgow, UK

**Keywords:** Coffee, Preeclampsia, Pregnancy, Systematic review, Meta-analysis

## Abstract

**Background:**

A growing body of evidence has documented unfavorable maternal outcomes attributed to excessive antenatal coffee consumption. Preeclampsia is one of the most common hypertensive disorders of pregnancy with several adverse maternal and neonatal outcomes. However, the association between antenatal coffee consumption and preeclampsia remains debatable. Herein, we performed a systematic review and meta-analysis of available evidence to investigate this association.

**Methods:**

After systematically reviewing PubMed and Scopus for eligible studies published until October 2023, we pooled the odds ratios (ORs) and their 95% confidence intervals (CIs) of preeclampsia for women who reported the highest versus the lowest frequencies of antenatal coffee consumption. We used the *I*^2^ statistic to measure heterogeneity across studies and the funnel plot asymmetry to assess publication bias.

**Results:**

This meta-analysis included seven retrospective studies (six case-control studies and one cross-sectional study) investigating 904 women with preeclampsia and 6,257 women without it. Combined, the highest frequencies of antenatal coffee consumption were associated with higher odds of preeclampsia: (pooled OR = 1.39, 95% CI: 1.03, 1.86), with a moderate heterogeneity across studies (*I*^2^ = 40.34% and p-value for heterogeneity = 0.122) and no publication bias (z = 0.610 and p-value for publication bias = 0.542). However, excluding the cross-sectional study, which contributed to 24.3% of the meta-analysis weight, left the association statistically non-significant: (pooled OR = 1.33, 95% CI: 0.91, 1.95; *I*^2^ = 44.59%). The association became even weaker after limiting the analysis to studies that excluded women with chronic hypertension: (pooled OR = 1.21, 95% CI: 0.77, 1.89; *I*^2^ = 41.64%) or after excluding studies with low quality: (pooled OR = 1.24, 95% CI: 0.70, 2.19; *I*^2^ = 65.79%).

**Conclusion:**

The association between antenatal coffee consumption and preeclampsia remains inconclusive. Future prospective cohort studies are needed to better investigate this association.

**Supplementary information:**

The online version contains supplementary material available at https://doi.org/10.1265/ehpm.24-00149.

## 1. Introduction

Preeclampsia is the most prevalent subtype of hypertensive disorders of pregnancy (HDP) with a prevalence of approximately 3–10% of all pregnancies [1–4]. In addition to hypertension, preeclampsia is characterized by coexisting proteinuria or generalized edema diagnosed after 20 weeks of gestation [1]. Given its adverse maternal and neonatal outcomes, preeclampsia is considered a leading cause of maternal and neonatal morbidity and mortality worldwide [1, 2]. Additionally, preeclampsia poses a huge financial burden. Based on its subsequent major maternal and perinatal morbidity and mortality (acute and long-term complications) and related maternal and perinatal programs, the total cost burden of preeclampsia in the US, according to 2012 claims, was $2.18 billion during the first 12 months of delivery ($1.03 billion for mothers and $1.15 billion for infants) [5]. Using electronic health records and billing data from a large regional integrated healthcare system in Pennsylvania, the maternal and infant healthcare costs in the preeclampsia cohort were triple that of the uncomplicated cohort [6].

However, preeclampsia is not inevitable and could be prevented by avoiding its risk factors [1, 2]. Evidence has suggested that excessive antenatal caffeine consumption could carry a higher risk of maternal and fetal complications [7, 8]. Despite these potential risks, pregnant women, especially those in the US and Europe, consume caffeine regularly [8–10]. Coffee, a heavily consumed beverage, is a major source of caffeine [11]. Caffeine may elevate blood pressure by antagonizing adenosine receptors, stimulating catecholamine release [12]. It also promotes renin release from the kidneys, leading to angiotensin II production [13]. Chronic caffeine use can impair endothelial function, reducing nitric oxide and increasing vascular resistance [14]. In addition, caffeine affects intracellular calcium signaling, enhancing smooth muscle contractility and vasoconstriction [15]. Together, these factors can elevate blood pressure.

The potential involvement of coffee consumption in the occurrence of preeclampsia remains debated. Some studies have investigated this association; however, they were limited by the small number of preeclampsia cases and the inconsistent findings [16–24]. Therefore, we performed a meta-analysis of available evidence to investigate the association between antenatal coffee consumption and preeclampsia.

## 2. Methods

We reported this meta-analysis according to the checklist of the Preferred Reporting Items for Systematic Reviews and Meta-Analysis (PRISMA) and Meta-analysis Of Observational Studies in Epidemiology (MOOSE) [25, 26].

### 2.1. Eligibility criteria

Our eligibility criteria were as follows: 1) the study had an observational design, 2) antenatal coffee consumption was the exposure, 3) preeclampsia was the outcome, and 4) the risk estimate or prevalence of preeclampsia for consuming the highest versus lowest frequencies of coffee during pregnancy was shown.

### 2.2. Information sources

PubMed and Scopus databases were independently searched by the first and second authors for potential studies published before October 1st, 2023.

### 2.3. Search strategy

We used the following search terms: (Coffee) AND ((Preeclampsia) OR (Pregnancy) OR (Hypertension)) (Supplementary Table 1). Furthermore, we manually searched the reference lists of the obtained studies to retrieve more studies. We set no limits regarding the publication year; however, we only considered studies published in English.

### 2.4. Study selection

**Fig. 1:**
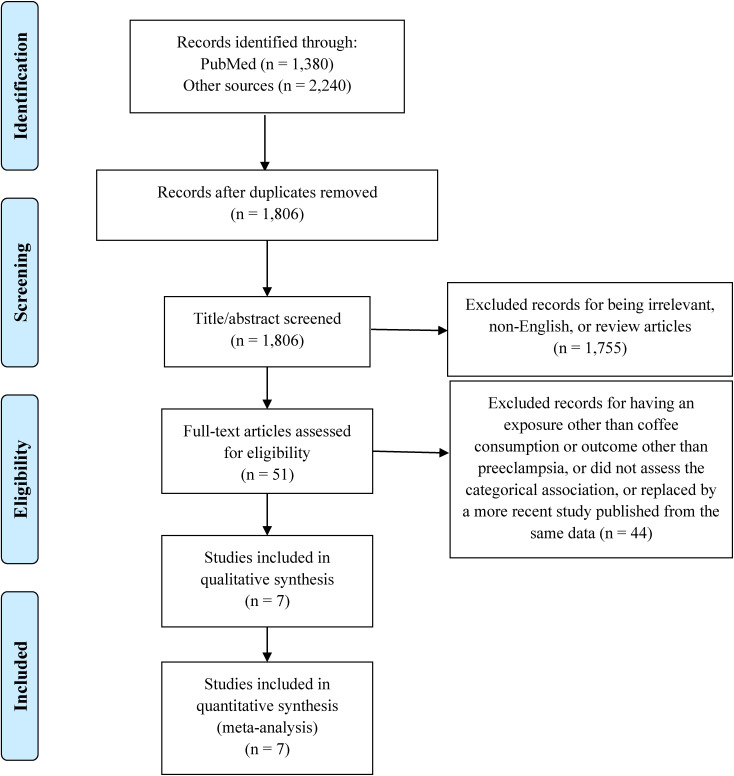
PRISMA flow diagram of the study selection process for the meta-analysis

We excluded duplicates, irrelevant studies, review articles, one article that did not categorize coffee consumption and assessed the linear association instead [23], and another study that investigated HDP as a whole rather than preeclampsia [24]. Eventually, we reached a shortlist of seven eligible studies for meta-analysis (Fig. 1).

### 2.5. Data extraction

The following data were extracted by the first and second authors from the selected studies: the last name of the first author, year of publication, study country, sample size, study design, coffee consumption categories, exclusion criteria, adjusted variables, and the odds ratios (ORs) with 95% confidence intervals (CIs) of the most adjusted models.

### 2.6. Assessment of risk of bias

The quality of studies and their risk of bias were assessed using the modified Newcastle–Ottawa Scale (NOS) in terms of case definition, representativeness of cases, selection of controls, the definition of controls, comparability, ascertainment of exposure, applying the same method of ascertainment for cases and controls, and nonresponse rate [27]. The first and second authors decided on the quality of the studies with disagreements solved by discussion.

### 2.7. Statistical analysis

The random-effects model was used to compute the pooled OR (95% CI) of preeclampsia among women who reported the highest versus lowest frequencies of antenatal coffee consumption [28]. We assessed statistical heterogeneity across studies using the *I*^2^ statistic [29] and publication bias using the regression test for funnel plot asymmetry [30]. We further stratified the results by several characteristics. The R-3.2.0 statistical package (Metafor: Meta-Analysis Package for R) was used for meta-analysis [31].

## 3. Results

**Fig. 2:**
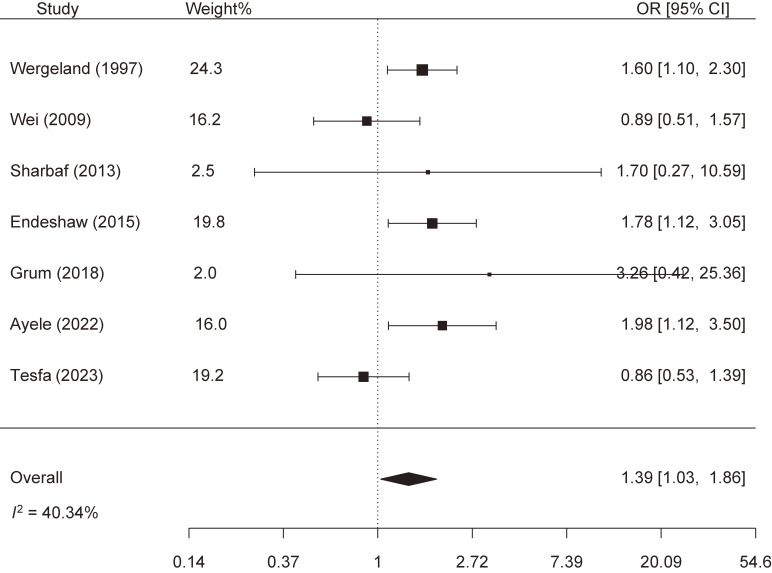
Meta-analysis of the association between antenatal coffee consumption and preeclampsia

**Fig. 3:**
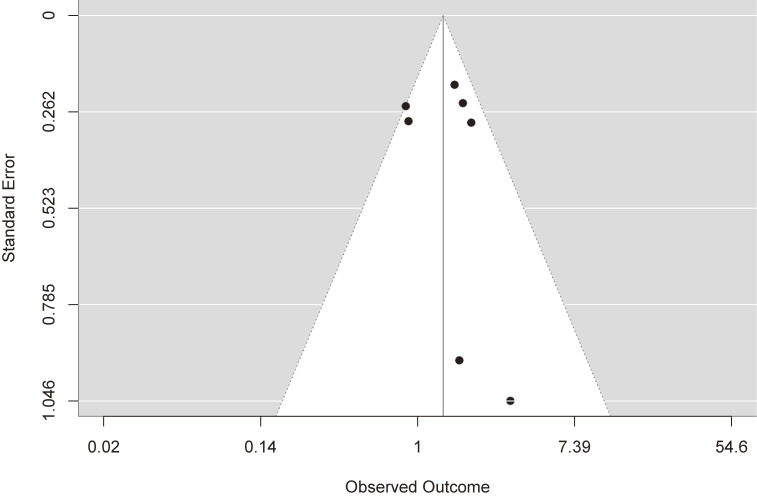
Funnel plot of the included studies

This meta-analysis included seven observational studies (six case-control studies and one cross-sectional study) investigating women from Norway, Canada, Iran, and Ethiopia. The studies included 904 women with preeclampsia and 6,257 women without it and were published between 1997 and 2023 (Table 1). Three studies showed higher odds of preeclampsia among those who reported the highest frequencies of antenatal coffee consumption [16, 19, 21], while the other four studies showed no association [17, 18, 20, 22]. Combined, women who reported the highest frequencies of antenatal coffee consumption showed increased odds of preeclampsia (pooled OR = 1.39, 95% CI: 1.03, 1.86) compared to those who reported the lowest frequencies. We detected moderate heterogeneity across studies, yet statistically non-significant (*I*^2^ = 40.34% and p-value for heterogeneity = 0.122) (Fig. 2). No publication bias was detected (z = 0.610 and p-publication bias = 0.542) (Fig. 3). Importantly, the association became statistically non-significant when the analysis was restricted to case-control studies: 1.33 (0.91, 1.95). Besides, the results showed obvious variations in the stratified analysis: 1.49 (0.92, 2.42) in the Ethiopian studies versus 1.24 (0.70, 2.19) in the Western studies; 1.45 (1.03, 2.05) in studies that adjusted for confounders versus 1.32 (0.68, 2.58) in studies that did not; 1.66 (1.23, 2.24) in studies that excluded women with chronic hypertension versus 1.21 (0.77, 1.89) in studies that did not; 1.50 (1.08, 2.09) in studies that did not define coffee consumption trimester versus 0.94 (0.55, 1.61) in studies that limited their analysis to coffee consumption during first trimester; and 1.49 (0.96, 2.29) in studies with low quality versus 1.24 (0.70, 2.19) in studies with average quality (Table 2). Based on the modified NOS criteria, two studies had average-quality scores and low risk of bias [16, 17], while five studies had low-quality scores and potential bias in terms of case representativeness, comparability, and ascertainment of coffee consumption [18–22] (Table 3).

**Table 1:** Summary of the studies included in the meta-analysis

**Study ID**	**Study design**	**Population**	**Drinking categories**	**Exclusion criteria**	**Adjusted variables**
Wergeland and Strand (1997) [16]	Retrospective Cross-sectional	Cases: 284 Controls: 5,104 Participants were recruited from maternity institutions in Norway (1989)	≤4 (Ref) and >4 cups/day	Multiparous	Age, height, body mass index, parity, education, smoking during pregnancy, and paid work
Wei et al. (2009) [17]	Retrospective Case-control	Cases: 92 Controls: 245 Participants were recruited within 48 hours after delivery from 4 hospitals in Quebec, Canada (2003–2006)	Never (Ref), 0–7, and ≥7 cups/week during the first 20 weeks of pregnancy	Age <18 years, multiparous, heart disorders, pregestational diabetes, HIV-positive serology, chronic hypertension, hypertension before 20 weeks of pregnancy, or gestational hypertension without proteinuria	Maternal age, body mass index, education, smoking, and history of abortion
Sharbaf et al. (2013) [18]	Retrospective Case-control	Cases: 40 Controls: 100 Participants were recruited within 48 hours after delivery from 2 hospitals in Tehran, Iran (2009–2010)	Never (Ref) and yes during the first trimester	Age <18 or >35 years, body mass index <19 or >22 kg/m^2^, multiparous, chronic hypertension, heart disorder, HIV positive serology, or history of intrauterine fetal death or abortion	NA
Endeshaw et al. (2015) [19]	Retrospective Case-control	Cases: 151 Controls: 302 Participants were recruited from the public health facilities of Bahir Dar city, Ethiopia (2014)	No (Ref) and daily	Staying <6 months in Bahir Dar city, Ethiopia	Maternal age, residence, mid-upper arm circumference, fruit and vegetable intake, folate intake, and anemia
Grum et al. (2018) [20]	Retrospective Case-control	Cases: 81 Controls: 162 Participants were recruited from 2 hospitals in Addis Ababa, Ethiopia (2015–2016)	No (Ref) and daily	Chronic hypertension or renal diseases	Parity, previous preeclampsia, nutritional counseling, pregnancy interval, and fruit and vegetable intake
Ayele and Tilahun (2022) [21]	Retrospective Case-control	Cases: 88 Controls: 176 Participants were recruited from the public health facilities of Debre Tabor Town, Ethiopia (2020–2021)	No (Ref) and yes	Chronic hypertension or renal diseases	NA
Tesfa et al. (2023) [22]	Retrospective Case-control	Cases: 168 Controls: 168 Participants were recruited from public hospitals in Bahir Dar city, Ethiopia (2020–2021)	No (Ref) and yes	Chronic hypertension, gestational hypertension, gestational age <20 weeks, or severely ill	NA

**Table 2:** Meta-analysis of the association between antenatal coffee consumption and preeclampsia stratified by certain characteristics

**Characteristics**		**Number of studies**	**OR (95% CI)**	***I* ^2^**
Design	Case-control	6	1.33 (0.91, 1.95)	44.59%
	Cross-sectional	1	1.60 (1.10, 2.30)	—

Region	West	2	1.24 (0.70, 2.19)	65.79%
	Ethiopia	4	1.49 (0.92, 2.42)	56.38%
	Iran	1	1.70 (0.27, 10.95)	—

Reference category for coffee consumption	Never	6	1.33 (0.91, 1.95)	44.59%
	Low amount	1	1.60 (1.10, 2.30)	—

Trimester of coffee consumption	First trimester	2	0.94 (0.55, 1.61)	0.00%
	Others/undefined	5	1.50 (1.08, 2.09)	43.95%

Adjustment for confounders	Yes	4	1.45 (1.03, 2.05)	31.87%
	No	3	1.32 (0.68, 2.58)	59.33%

Excluding women with chronic hypertension	Yes	5	1.21 (0.77, 1.89)	41.64%
	No	2	1.66 (1.23, 2.24)	0.00%

Overall quality	Average	2	1.24 (0.70, 2.19)	65.79%
	Low	5	1.49 (0.96, 2.29)	42.15%

**Table 3:** Risk of bias assessment using the Newcastle-Ottawa Quality Assessment Scale

**Item**	**Wergeland and Strand [** 16 **]**	**Wei et al. [** 17 **]**	**Sharbaf et al. [** 18 **]**	**Endeshaw et al. [** 19 **]**	**Grum et al. [** 20 **]**	**Ayele and Tilahun [** 21 **]**	**Tesfa et al. [** 22 **]**
Case definition	*	*	*	*	*	*	*
Representativeness of cases	*	*	-	-	-	-	-
Selection of controls	*	*	*	*	*	*	*
Definition of controls	*	*	*	*	*	*	*
Comparability	*	**	-	-	-	-	-
Ascertainment of exposure	*	*	-	-	-	-	-
The same method of ascertainment for cases and controls	*	*	*	*	*	*	*
Nonresponse rate	*	-	-	-	-	-	-
Overall (total number of asterisks)	8	8	4	4	4	4	4

The possible overall scores range between 0 and 9

## 4. Discussion

This meta-analysis included six case-control studies and one cross-sectional study. When combined, women with the highest frequencies of antenatal coffee consumption showed higher odds of preeclampsia and moderate heterogeneity across studies. However, excluding the cross-sectional study, which contributed to one-fourth of the meta-analysis weight, left the association statistically non-significant. Furthermore, the association became statistically non-significant in studies with average quality, studies that excluded women with chronic hypertension, and studies that limited coffee consumption analysis to the first trimester. The inconclusive link between antenatal coffee consumption and preeclampsia is mostly due to differences in study populations, inconsistent designs, varying coffee consumption thresholds, and potential confounding factors, making it difficult to establish a conclusive relationship.

The mechanisms underlying the association between antenatal coffee consumption and preeclampsia are uncertain. Yet, it could be suggested that caffeine, a leading active element in coffee that freely passes the placenta, may increase catecholamines, subsequently elevating blood pressure [32]. The Generation R Study showed a positive association between antenatal caffeine consumption and systolic blood pressure [33]. A recent meta-analysis of four observational studies showed that excessive antenatal tea consumption, another major source of caffeine, was associated with increased HDP (OR = 1.16, 95% CI: 1.01, 1.33) [34]. The World Health Organization, therefore, recommends that pregnant women’s daily caffeine consumption should stay below 200 mg and not exceed 300 mg [35].

In contrast, van der Hoeven et al. showed that Dutch women who consumed coffee during pregnancy, assessed as continuous variables, did not experience higher rates of preeclampsia: OR (95% CI) for cup/day = 0.81 (0.46, 1.44), pregnancy-induced hypertension: OR (95% CI) for cup/day = 0.94 (0.77, 1.16), or HELLP (Hemolysis, Elevated Liver enzymes, and Low Platelets): OR (95% CI) for cup/day = 1.30 (0.70, 2.43) [23]. However, the Dutch study adjusted their results only for age and smoking behavior, suggesting that several factors might have confounded the results. Additionally, it could be speculated that the hazardous impacts of coffee consumption could be detected with higher doses only. Since the analysis of the Dutch study was based on a one-unit change, the hypertensive effects of excessive coffee consumption were unlikely to be detected. Similarly, Kawanishi et al. showed that antenatal consumption of coffee was not associated with HDP among Japanese women: OR (95% CI) = 0.89 (0.72, 1.09) for ≥two cups/day versus none [24]. However, most participants in the Japanese study consumed caffeine far below the amounts described in Western studies. A cohort study using data from a longitudinal pregnancy cohort study from the National Institute of Child Health and Human Development (NICHD) showed an increasing trend of preeclampsia risk across the increasing doses of caffeinated beverage intake during the past week, yet this trend was statistically non-significant due to the limited number of participants: ORs (95% CIs) = 0.99 (0.59–1.64) for 1–100 mg/day, 1.23 (0.52–2.89) for 101–200 mg/day, and 2.74 (0.56–13.30) for >200 mg/day [36]. These findings indicate that antenatal coffee consumption could contribute to preeclampsia risk only at high doses.

Apart from pregnant women, the association between coffee consumption and hypertension risk remains controversial. In a study including 24,710 Finnish men and women, aged 25–64 years, coffee drinking seemed to increase the risk of hypertension: hazard ratios (95% CIs) of incident hypertension for drinking ≤one, two to three, four to five, six to seven, and ≥eight cups/day were 1.00, 1.29 (1.09, 1.54), 1.26 (1.06, 1.49), 1.24 (1.04, 1.48), and 1.14 (0.94, 1.37), respectively (p-value for trend = 0.024) [37]. In the Nurses’ Health Studies (NHSs) I and II of 155,594 US women, aged 30–55 years, coffee drinking seemed to reduce the risk of hypertension: hazard ratios (95% CIs) of incident hypertension for drinking <one, one, two to three, four to five, and ≥six cups/day) in the NHSI were 1.00, 1.06 (1.01, 1.10), 1.00 (0.97, 1.04), 0.93 (0.88, 0.99), and 0.88 (0.80, 0.98), respectively (p-value for trend = 0.020) and the NHS II were 1.00, 1.06 (1.01, 1.13), 1.00 (0.95, 1.04), 0.91 (0.84, 0.98), and 0.91 (0.80, 1.04), respectively (p-value for trend = 0.030) [38].

The association between coffee consumption and blood pressure could be modified by coffee consumption habits. A randomized crossover trial among 38 habitual and 39 non-habitual coffee drinkers showed that espresso consumption resulted in blood pressure elevation in non-habitual drinkers but not in habitual drinkers [39]. In addition, a randomized controlled trial of 52 habitual and 84 non-habitual drinkers showed a decrease in blood pressure among habitual drinkers and an increase in blood pressure among non-habitual drinkers [40].

Antenatal coffee consumption could also be associated with other maternal medical conditions. For example, a meta-analysis of 34 studies (18 cohort studies and 16 case-control studies) demonstrated that caffeine intake during pregnancy was associated with a higher risk of pregnancy loss; an increase of a cup of coffee/day during pregnancy was associated with a 3% increased risk of pregnancy loss [41]. Other studies showed that caffeine consumption during pregnancy was associated with intrauterine growth retardation, low birth weight, and spontaneous abortion [42–44].

This meta-analysis has several limitations that should be considered. First, only seven studies were eligible for inclusion in the meta-analysis. The limited number of studies made it difficult to further stratify the meta-analysis by potential confounders to obtain a better understanding of the possible impact of controlling for certain confounders on the overall conclusion. For the same reason, the results stratified by various variables should be interpreted cautiously. Second, the included studies used different cut-offs and definitions for antenatal coffee consumption, suggesting misclassification. Third, the retrospective design of the included studies may have hidden a recall bias risk. Fourth, we confined the meta-analysis to preeclampsia because only one study investigated HDP as a whole [24]. Caffeine exposure during pregnancy might have different effects on HDP types. For example, a recent meta-analysis showed that antenatal caffeine consumption was not associated with the risk of gestational hypertension (OR = 0.99, 95% CI: 0.90, 1.08) but showed a modest increase, however statistically non-significant, in the risk of preeclampsia (OR = 1.13, 95% CI: 0.97, 1.31) [45]. Fifth, all included studies in this meta-analysis did not control their results for the frequency of coffee consumption before pregnancy. Previous trials showed that coffee consumption resulted in elevated blood pressure in non-habitual drinkers but not in habitual drinkers [39, 40]. Sixth, although this meta-analysis included women from different races, East Asian women, who typically consume less caffeine during pregnancy than pregnant women in Western populations [24], were not represented. Seventh, none of the included studies examined the association with decaffeinated coffee to understand whether the association between antenatal coffee consumption and preeclampsia is mainly explained by caffeine intake. Eighth, it could be speculated, due to the observational design of the included studies, that some confounders might have gone undetected. Coffee consumption is closely associated with smoking, smoking urges, and subjective smoking reinforcement in daily life [46], and smoking can influence preeclampsia [47, 48]. Besides, coffee consumption could reduce sleep duration and worsen sleep quality [49], and maternal sleep disorders might contribute to the development of preeclampsia [50]. Additionally, many studies did not adjust their results for other important preeclampsia risk factors, such as age, multiple pregnancies, short intervals between pregnancies, obesity, hypertension, diabetes, chronic kidney disease, and autoimmune disorders [1, 51].

In summary, the association between antenatal coffee consumption and preeclampsia, based on available evidence, remains inconclusive. This uncertainty likely stems from variability in study designs, confounding factors, and differences in populations. Inconsistent methodologies, such as varying measures of coffee intake and timing during pregnancy, combined with potential confounders like diet and lifestyle, make it difficult to draw clear conclusions. Additionally, the biological mechanisms by which caffeine influences preeclampsia are not fully understood, contributing to the mixed results across studies. Therefore, well-designed prospective cohort studies are needed to better investigate this association, establish causality, and explore potential dose-response relationships. Research on the effects of specific coffee types, caffeine content, and habitual consumption on maternal health outcomes is necessary for tailored recommendations for pregnant women. Future studies to clarify the biological mechanisms behind this association are also warranted.

## Abbreviations

CI: Confidence interval
HELLP: Hemolysis, Elevated Liver enzymes, and Low Platelets
HDP: Hypertensive disorders of pregnancy
NHS: Nurses’ Health Study
NICHD: National Institute of Child Health and Human Development
NOS: Newcastle–Ottawa Scale
OR: Odds ratio

## Supplementary information

**Additional file 1:** **Supplementary Table 1:** PubMed search strategy.

## Declarations

### Availability of data and materials

No original datasets were created.

## References

[1] Fox R, Kitt J, Leeson P, Aye CYL, Lewandowski AJ. Preeclampsia: risk factors, diagnosis, management, and the cardiovascular impact on the offspring. J Clin Med. 2019;8(10):1625. 31590294 10.3390/jcm8101625 PMC6832549

[2] Jeyabalan A. Epidemiology of preeclampsia: impact of obesity. Nutr Rev. 2013;71 Suppl 1(0 1):S18–25. 24147919 10.1111/nure.12055 PMC3871181

[3] Abalos E, Cuesta C, Grosso AL, Chou D, Say L. Global and regional estimates of preeclampsia and eclampsia: a systematic review. Eur J Obstet Gynecol Reprod Biol. 2013;170(1):1–7. 23746796 10.1016/j.ejogrb.2013.05.005

[4] Yang Y, Le Ray I, Zhu J, Zhang J, Hua J, Reilly M. Preeclampsia prevalence, risk factors, and pregnancy outcomes in Sweden and China. JAMA Netw Open. 2021;4(5):e218401. 33970258 10.1001/jamanetworkopen.2021.8401 PMC8111481

[5] Stevens W, Shih T, Incerti D, . Short-term costs of preeclampsia to the United States health care system. Am J Obstet Gynecol. 2017;217(3):237–48.e16. 28708975 10.1016/j.ajog.2017.04.032

[6] Hao J, Hassen D, Hao Q, . Maternal and infant health care costs related to preeclampsia. Obstet Gynecol. 2019;134(6):1227–33. 31764733 10.1097/AOG.0000000000003581 PMC6882523

[7] Qian J, Chen Q, Ward SM, Duan E, Zhang Y. Impacts of caffeine during pregnancy. Trends Endocrinol Metab. 2020;31(3):218–27. 31818639 10.1016/j.tem.2019.11.004 PMC7035149

[8] James JE. Maternal caffeine consumption and pregnancy outcomes: a narrative review with implications for advice to mothers and mothers-to-be. BMJ Evid Based Med. 2021;26(3):114–5. 10.1136/bmjebm-2020-111432 PMC8165152 32843532

[9] Román-Gálvez MR, Martín-Peláez S, Hernández-Martínez L, . Caffeine intake throughout pregnancy, and factors associated with non-compliance with recommendations: a cohort study. Nutrients. 2022;14(24):5384. 36558543 10.3390/nu14245384 PMC9785327

[10] Weng X, Odouli R, Li DK. Maternal caffeine consumption during pregnancy and the risk of miscarriage: a prospective cohort study. Am J Obstet Gynecol. 2008;198(3):279.e1–8. 10.1016/j.ajog.2007.10.803 18221932

[11] Drewnowski A, Rehm CD. Sources of caffeine in diets of US children and adults: trends by beverage type and purchase location. Nutrients. 2016;8(3):154. 26978391 10.3390/nu8030154 PMC4808882

[12] Smith A, Brice C, Nash J, Rich N, Nutt DJ. Caffeine and central noradrenaline: effects on mood, cognitive performance, eye movements and cardiovascular function. J Psychopharmacol. 2003;17(3):283–92. 14513920 10.1177/02698811030173010

[13] Tofovic SP, Kusaka H, Pfeifer CA, Jackson EK. Central effects of caffeine on renal renin secretion and norepinephrine spillover. J Cardiovasc Pharmacol. 1996;28(2):302–13. 8856488 10.1097/00005344-199608000-00018

[14] Echeverri D, Montes FR, Cabrera M, Galán A, Prieto A. Caffeine’s vascular mechanisms of action. Int J Vasc Med. 2010;2010:834060. 21188209 10.1155/2010/834060 PMC3003984

[15] Kramer RH, Mokkapatti R, Levitan ES. Effects of caffeine on intracellular calcium, calcium current and calcium-dependent potassium current in anterior pituitary GH3 cells. Pflugers Arch. 1994;426(1–2):12–20. 8146014 10.1007/BF00374665

[16] Wergeland E, Strand K. Working conditions and prevalence of pre-eclampsia, Norway 1989. Int J Gynaecol Obstet. 1997;58(2):189–96. 9252254 10.1016/s0020-7292(97)00083-0

[17] Wei SQ, Xu H, Xiong X, Luo ZC, Audibert F, Fraser WD. Tea consumption during pregnancy and the risk of pre-eclampsia. Int J Gynaecol Obstet. 2009;105(2):123–6. 19195655 10.1016/j.ijgo.2008.12.003

[18] Sharbaf FR, Dehghanpour P, Shariat M, Dalili H. Caffeine consumption and incidence of hypertension in pregnancy. J Family Reprod Health. 2013;7(3):127–30.

[19] Endeshaw M, Abebe F, Bedimo M, Asart A. Diet and pre-eclampsia: a prospective multicentre case-control study in Ethiopia. Midwifery. 2015;31(6):617–24. 25862389 10.1016/j.midw.2015.03.003

[20] Grum T, Hintsa S, Hagos G. Dietary factors associated with preeclampsia or eclampsia among women in delivery care services in Addis Ababa, Ethiopia: a case control study. BMC Res Notes. 2018;11(1):683. 30285827 10.1186/s13104-018-3793-8 PMC6167851

[21] Ayele AD, Tilahun ZA. Determinants of pre-eclampsia among women attending delivery services in public health institutions of Debre Tabor Town: a case-control study. Reprod Health. 2022;19(1):157. 35804383 10.1186/s12978-022-01463-1 PMC9270738

[22] Tesfa E, Munshea A, Nibret E, Gizaw ST. Determinants of pre-eclampsia among pregnant women attending antenatal care and delivery services at Bahir Dar public hospitals, northwest Ethiopia: A case-control study. Health Sci Rep. 2023;6(7):e1440. 37519426 10.1002/hsr2.1440 PMC10372301

[23] van der Hoeven T, Browne JL, Uiterwaal CSPM, van der Ent CK, Grobbee DE, Dalmeijer GW. Antenatal coffee and tea consumption and the effect on birth outcome and hypertensive pregnancy disorders. PLoS One. 2017;12(5):e0177619. 28520809 10.1371/journal.pone.0177619 PMC5433714

[24] Kawanishi Y, Kakigano A, Kimura T, . Hypertensive disorders of pregnancy in relation to coffee and tea consumption: The Japan Environment and Children’s Study. Nutrients. 2021;13(2):343. 33498916 10.3390/nu13020343 PMC7912571

[25] Moher D, Liberati A, Tetzlaff J, Altman DG; PRISMA Group. Preferred reporting items for systematic reviews and meta-analyses: the PRISMA statement. PLoS Med. 2009;6:e1000097. 19621072 10.1371/journal.pmed.1000097 PMC2707599

[26] Brooke BS, Schwartz TA, Pawlik TM. MOOSE Reporting Guidelines for Meta-analyses of Observational Studies. JAMA Surg. 2021;156:787–8. 33825847 10.1001/jamasurg.2021.0522

[27] Stang A. Critical evaluation of the Newcastle-Ottawa scale for the assessment of the quality of nonrandomized studies in meta-analyses. Eur J Epidemiol. 2010;25:603–5. 20652370 10.1007/s10654-010-9491-z

[28] DerSimonian R, Laird N. Meta-analysis in clinical trials. Control Clin Trials. 1986;7(3):177–88. 3802833 10.1016/0197-2456(86)90046-2

[29] Higgins J, Thompson S, Deeks J, Altman D. Measuring inconsistency in meta-analyses. BMJ. 2003;327:557–60. 12958120 10.1136/bmj.327.7414.557 PMC192859

[30] Egger M, Davey Smith G, Schneider M, Minder C. Bias in meta-analysis detected by a simple, graphical test. BMJ. 1997;315:629–34. 9310563 10.1136/bmj.315.7109.629 PMC2127453

[31] Viechtbauer W. Conducting meta-analyses in R with the metafor package. J Stat Softw. 2010;36(10):1–48.

[32] Smith A, Brice C, Nash J, Rich N, Nutt DJ. Caffeine and central noradrenaline: effects on mood, cognitive performance, eye movements and cardiovascular function. J Psychopharmacol. 2003;17(3):283–92. 14513920 10.1177/02698811030173010

[33] Bakker R, Steegers EA, Raat H, Hofman A, Jaddoe VW. Maternal caffeine intake, blood pressure, and the risk of hypertensive complications during pregnancy. The Generation R Study. Am J Hypertens. 2011;24(4):421–8. 21164492 10.1038/ajh.2010.242

[34] Arafa A, Sheerah HA, Alzaydan OK, Sabr Y. The association between antenatal tea drinking and hypertensive disorders of pregnancy: a systematic review and meta-analysis. Epidemiologia. 2024;5(2):200–10. 38804341 10.3390/epidemiologia5020014 PMC11130964

[35] WHO Guidelines Approved by the Guidelines Review Committee. WHO recommendations on antenatal care for a positive pregnancy experience. Geneva: World Health Organization 2016. https://www.who.int/publications/i/item/9789241549912. Accessed on 18 March 2023.

[36] Hinkle SN, Gleason JL, Yisahak SF, . Assessment of caffeine consumption and maternal cardiometabolic pregnancy complications. JAMA Netw Open. 2021;4(11):e2133401. 34748005 10.1001/jamanetworkopen.2021.33401 PMC8576579

[37] Hu G, Jousilahti P, Nissinen A, Bidel S, Antikainen R, Tuomilehto J. Coffee consumption and the incidence of antihypertensive drug treatment in Finnish men and women. Am J Clin Nutr. 2007;86(2):457–64. 17684219 10.1093/ajcn/86.2.457

[38] Winkelmayer WC, Stampfer MJ, Willett WC, Curhan GC. Habitual caffeine intake and the risk of hypertension in women. JAMA. 2005;294(18):2330–5. 16278361 10.1001/jama.294.18.2330

[39] Zimmermann-Viehoff F, Thayer J, Koenig J, Herrmann C, Weber CS, Deter HC. Short-term effects of espresso coffee on heart rate variability and blood pressure in habitual and non-habitual coffee consumers--a randomized crossover study. Nutr Neurosci. 2016;19(4):169–75. 25850440 10.1179/1476830515Y.0000000018

[40] Hara A, Ohide H, Miyagawa K, . Acute effects of caffeine on blood pressure and heart rate in habitual and non-habitual coffee consumers: a randomized, double-blind, placebo-controlled study. Jpn J Pharm Health Care Sci. 2014;40(7):383–8.

[41] Jafari A, Naghshi S, Shahinfar H, . Relationship between maternal caffeine and coffee intake and pregnancy loss: a grading of recommendations assessment, development, and evaluation-assessed, dose-response meta-analysis of observational studies. Front Nutr. 2022;9:886224. 36017225 10.3389/fnut.2022.886224 PMC9396037

[42] CARE Study Group. Maternal caffeine intake during pregnancy and risk of fetal growth restriction: a large prospective observational study. BMJ. 2008;337:a2332. 18981029 10.1136/bmj.a2332 PMC2577203

[43] Chen LW, Wu Y, Neelakantan N, Chong MF, Pan A, van Dam RM. Maternal caffeine intake during pregnancy is associated with risk of low birth weight: a systematic review and dose-response meta-analysis. BMC Med. 2014;12:174. 25238871 10.1186/s12916-014-0174-6 PMC4198801

[44] Cnattingius S, Signorello LB, Annerén G, . Caffeine intake and the risk of first-trimester spontaneous abortion. N Engl J Med. 2000;343(25):1839–45. 11117975 10.1056/NEJM200012213432503

[45] Chen B, Zhang M, He Y, . The association between caffeine exposure during pregnancy and risk of gestational hypertension/preeclampsia: a meta-analysis and systematical review. J Obstet Gynaecol Res. 2022;48(12):3045–55. 36156331 10.1111/jog.15445 PMC10087308

[46] Treloar HR, Piasecki TM, McCarthy DE, Baker TB. Relations among caffeine consumption, smoking, smoking urge, and subjective smoking reinforcement in daily life. J Caffeine Res. 2014;4(3):93–9. 25229011 10.1089/jcr.2014.0007 PMC4158991

[47] Lewandowska M, Więckowska B. The influence of various smoking categories on the risk of gestational hypertension and pre-eclampsia. J Clin Med. 2020;9(6):1743. 32512866 10.3390/jcm9061743 PMC7356904

[48] Karumanchi SA, Levine RJ. How does smoking reduce the risk of preeclampsia? Hypertension. 2010;55(5):1100–1. 20231524 10.1161/HYPERTENSIONAHA.109.148973 PMC2855389

[49] Clark I, Landolt HP. Coffee, caffeine, and sleep: a systematic review of epidemiological studies and randomized controlled trials. Sleep Med Rev. 2017;31:70–8. 26899133 10.1016/j.smrv.2016.01.006

[50] Georgiou N, Fasoulakis Z, Theodora M, . Association of pregestational maternal sleeping disorders and preeclampsia: a retrospective cohort study and review of the literature. Cureus. 2019;11(3):e4338. 31187003 10.7759/cureus.4338 PMC6541156

[51] English FA, Kenny LC, McCarthy FP. Risk factors and effective management of preeclampsia. Integr Blood Press Control. 2015;8:7–12. 25767405 10.2147/IBPC.S50641 PMC4354613

