# Supplementary material for: The association between antenatal coffee consumption and preeclampsia: a systematic review and meta-analysis
Source: Environ Health Prev Med. 2024 Sep 21;29:49. doi: 10.1265/ehpm.24-00149 (PMC11446635; doi:10.1265/ehpm.24-00149)
Supplement: Supplementary file 1 — Additional file 1: Supplementary Table 1: PubMed search strategy. [file ehpm-29-049-s001.docx]

**Supplementary Table 1: PubMed search strategy**

| Search: (Coffee) AND ((Preeclampsia) OR (Pregnancy) OR (Hypertension))  ("coffee"[MeSH Terms] OR "coffee"[All Fields] OR "coffee s"[All Fields] OR "coffees"[All Fields]) AND ("pre eclampsia"[MeSH Terms] OR "pre eclampsia"[All Fields] OR "preeclampsia"[All Fields] OR ("pregnancy"[MeSH Terms] OR "pregnancy"[All Fields] OR "pregnancies"[All Fields] OR "pregnancy s"[All Fields]) OR ("hypertense"[All Fields] OR "hypertension"[MeSH Terms] OR "hypertension"[All Fields] OR "hypertension s"[All Fields] OR "hypertensions"[All Fields] OR "hypertensive"[All Fields] OR "hypertensive s"[All Fields] OR "hypertensives"[All Fields])) |
| --- |
| Translations  Coffee: "coffee"[MeSH Terms] OR "coffee"[All Fields] OR "coffee's"[All Fields] OR "coffees"[All Fields]  Preeclampsia: "pre-eclampsia"[MeSH Terms] OR "pre-eclampsia"[All Fields] OR "preeclampsia"[All Fields]  Pregnancy: "pregnancy"[MeSH Terms] OR "pregnancy"[All Fields] OR "pregnancies"[All Fields] OR "pregnancy's"[All Fields]  Hypertension: "hypertense"[All Fields] OR "hypertension"[MeSH Terms] OR "hypertension"[All Fields] OR "hypertension's"[All Fields] OR "hypertensions"[All Fields] OR "hypertensive"[All Fields] OR "hypertensive's"[All Fields] OR "hypertensives"[All Fields] |
